# Altered Pore Composition and Flexibility in a Deafness-Associated TMC1 Variant: Insights from Molecular Dynamics Simulations

**DOI:** 10.1021/acschemneuro.5c00546

**Published:** 2025-11-25

**Authors:** Davide Zamboni, Valerio Marino, Anna Avesani, Giuditta Dal Cortivo, Gianluca Lattanzi, Daniele Dell’Orco

**Affiliations:** † Department of Physics, 19034 University of Trento, Via Sommarive 14, I-38123 Trento, Italy; ‡ Department of Neurosciences, Biomedicine and Movement Sciences, Section of Biological Chemistry, 19051 University of Verona, Strada Le Grazie 8, I-37134 Verona, Italy; § INFN-TIFPA, Trento Institute for Fundamental Physics and Applications, I- 38123 Trento, Italy

**Keywords:** transmembrane channel-like protein 1 (TMC1), deafness-associated mutant M654V, membrane coupling, pore structure

## Abstract

Transmembrane channel-like protein 1 (TMC1) forms the pore of the mechanotransduction channel in cochlear and vestibular hair cells, converting mechanical stimuli from sound and head movements into electrochemical signals. Recent evidence supports a dimeric structure for TMC1, with each monomer harboring an independent ion-conducting pore. The p.(M654V) variant, in which methionine 654 is substituted with valine, is associated with non-syndromic autosomal recessive deafness. In the present work, we used molecular dynamics (MD) simulations to compare the structural and biophysical properties of the wild-type and M654V-TMC1 variants, providing atomistic-level insights into subtle alterations in the mechanotransduction system. Our analysis reveals specific alterations in pore size, lipid composition of the pore walls, and the electrostatic environment. The results suggest that the two monomers function independently and underscore the critical role of lipids in shaping the pore architecture. Potential molecular mechanisms of M654V-associated pathogenicity include disrupted local interactions between transmembrane α-helices and residue 654, leading to reduced pore flexibility, a shifted choke point, and fewer lipid molecules incorporated into the pore walls. These findings provide mechanistic insights into TMC1 function and its impairment in deafness-associated variants.

## Graphical abstract

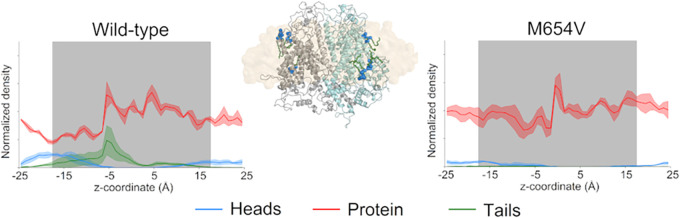

## 1 Introduction

The sense of hearing in mammals relies on the ability of specialized cells in the organ of Corti, the inner ear hair cells, to convert sound-induced mechanical stimuli into electrochemical signals through a complex biochemical cascade known as mechanotransduction. This process begins with the coordinated motion of the hair bundle in response to sound, facilitated by extracellular filaments called tip-links that connect the stereocilia.^1^ This motion induces structural remodeling of ion channels located near the lower end of the tip-links at the tips of the stereociliary rows, ultimately altering the ion conductivity. The molecular components of mechanotransduction and their precise roles have long been debated, as investigations have largely depended on genetic studies of deafness-associated mutations.^2,3^ Recent evidence suggests that the mechanotransduction complex is composed of the mechanically gated ion channel transmembrane channel-like protein 1 (TMC1), along with its key interacting partners: CIB2 (Calcium and Integrin Binding Protein 2), TMIE (Transmembrane Inner Ear protein), and possibly PCDH15 (protocadherin-15) as its key interacting partners.^4–9^ However, a direct biochemical characterization of the entire complex remains elusive due to low *in situ* expression levels of mammalian TMC1 and its mislocalization in heterologous expression systems.^10,11^ Moreover, gaining information at the atomistic level on signal transduction systems, particularly on mechanotransduction, is crucial for setting the basis for the future development of effective therapies for currently incurable hereditary diseases.

All-atom molecular dynamics (MD) simulations provide valuable insights into key structural features of biomolecular systems, often providing a dynamic mechanistic description that complements the static picture offered by experimental techniques, which are particularly challenging in the case of membrane proteins. TMC1 is a dimer, in which each monomer consists of 10 transmembrane (TM) α-helices (namely, TM1, residues 180–220, TM2, residues 280–320, TM3, residues 350–380, TM4, residues 402–427, TM5, residues 435–465, TM6, residues 517–552, TM7, residues 574–592, TM8, residues 595–620, TM9, residues 635–665, and TM10, residues 700–730) and hosts a peripheral, lipid-facing ion conduction pathway.

In this study, we performed >1.5 μs MD simulations of wild-type (wt) TMC1 and its M654V missense variant^3^ (Figure 1), which is associated with dominant non-syndromic hearing loss. The analysis of the dynamic structural features of the TMC1 wt pore revealed structural properties that may be essential for proper ion conduction during mechanotransduction. Comparison with the pathogenic variant highlighted key alterations that could underlie impaired function. Moreover, the observed differences between the wt and mutant proteins may help define the fundamental features that enable the mechanotransduction complex to finely regulate ion currents in response to a wide range of stimuli.

**Figure 1:**
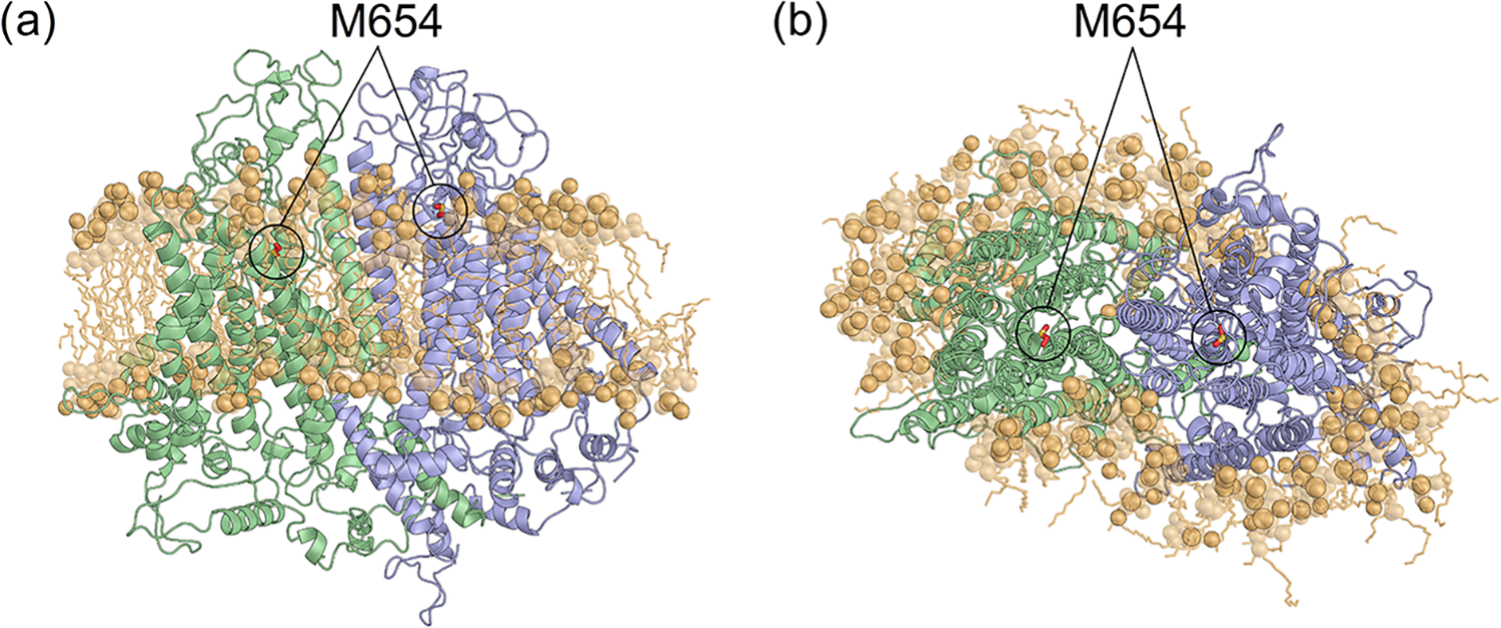
(a) Lateral and (b) top view of the three-dimensional structure of the TMC1 dimer. The protein is represented as a cartoon with monomer A in green and monomer B in blue, and residue M654 is shown as red sticks with S atoms in yellow. Lipids within 4 Å from the transmembrane helices are shown in transparency with heads represented as spheres and tails as sticks colored in light orange.

## 2 Methods

### 2.1 Molecular Modeling and Molecular Dynamics Simulations Setup

#### 2.1.1 Starting Systems

The coordinates of the starting structure were taken from ref 12, in which Cys-mutagenesis experiments and cryo-EM micrographs were consistent with electrophysiological recordings aimed at probing pore composition and permeability. The structure was preprocessed using the “Protein preparation” pipeline in Maestro, which assigned bond orders based on the Chemical Components Dictionary database (www.pdb.org, wwPDB Foundation, Piscataway, NJ, USA) and added hydrogen atoms. The protonation states of heteroatoms and ionizable residues at pH 7.5 were then predicted using Epik^13^ and PROPKA,^14^ respectively, followed by hydrogen bond assignment and optimization, considering crystal symmetry for protomer–protomer interactions. Subsequently, the protein structure was energy-minimized using OPLS4 force field (Schrödinger, New York, USA) with the Root-Mean-Square Deviation (RMSD) of heavy atoms constrained to 0.3 Å. The M654V variant was introduced in both TMC1 chains via *in silico* mutagenesis using Bioluminate’s Residue scanning tool (Schrödinger, New York, USA), selecting the most favorable rotamer, resulting in a homozygous dimer, which was then processed through the same “Protein preparation” pipeline as the wild type.

The CHARMM-GUI web server^15^ was used to embed the modeled structure of the two TMC1 variants in a 100% POPC (1-palmitoyl-2-oleoyl-*sn*-glycero-3-phosphocholine) lipid bilayer and solvate them in TIP3P water. Each system was then supplemented with 150 mM KCl and neutralized, yielding final systems of ∼400.000 and ∼405.000 atoms, respectively, contained within simulation boxes of 145.1 Å × 145.1 Å × 203.3 Å (*x*-, *y*-, and *z*-axes) and 145.0 Å × 145.0 Å × 206.0 Å for the TMC1 wt and M654V, respectively. Both systems were simulated using GROMACS 2019.6 ^16^ with the CHARMM36m force field.^17^ Long-range Coulombic interactions were computed using the Particle Mesh Ewald (PME)^18^ algorithm with a cutoff of 12 Å, a grid spacing of 1.2 Å, and fourth-order spline interpolation, while short-ranged van der Waals interactions were smoothly switched off using a force-switch function in the 10–12 Å range. Finally, the LINCS^19^ algorithm was applied to constrain covalent bond lengths involving hydrogen atoms.

#### 2.1.2 Minimization

The energy of both systems was minimized using the steepest descent algorithm to eliminate steric clashes, followed by two NVT and four NPT equilibration steps (see Supporting Table 1 for energy restraints), in accordance with the CHARMM-GUI relaxation protocol.

#### 2.1.3 NVT Equilibration

NVT equilibration was performed at 310 K using the velocity rescaling thermostat^21^ with a coupling time constant of 1.0 ps, in two consecutive 125 ps steps. In the first step, position restraints were applied to the heavy atoms of the TMC1 backbone (9.56 kcal/mol Å^2^) and side chains (4.78 kcal/mol Å^2^), while for POPC molecules, the motion along the *z*-axis of phosphorus atom was restrained (2.39 kcal/mol Å^2^), along with the dihedral angle formed by C1, C2, C3 and O21 (2.39 kcal/mol Å^2^). In the second NVT equilibration step, restraints on protein atoms were halved, while those on the POPC atoms were reduced to 1 kcal/mol Å^2^. During these equilibration steps, the temperature (Supporting Figure S1) and average solvent-accessible area per POPC molecule (Supporting Figure S2) remained stable.

#### 2.1.4 NPT Equilibration

NPT equilibration was conducted at constant pressure (1 atm) using the Berendsen barostat^22^ with semi-isotropic coupling and a time constant of 5 ps. The equilibration process consisted of four steps: (i) 125 ps (time step = 1 fs); (ii) and (iii) 500 ps (time step = 2 fs); (iv) 10 ns (time step = 2 fs). Position restraints were gradually reduced and eventually removed for all atoms. System density (Supporting Figure S3) and average solvent-accessible area per POPC molecule (Supporting Figure S4) remained stable during the equilibration steps, particularly during the last 6 ns, indicating that the systems had reached equilibrium.

#### 2.1.5 Production Runs

MD simulations were performed in the NPT ensemble at 310 K and 1 atm using the Nosè-Hoover thermostat^23,24^ (coupling period = 1 ps) and the Parrinello–Rahman barostat (coupling period = 5 ps).^25^ Three independent >500 ns trajectories were generated for each system, specifically 568.4 ns, 568.2 ns, and 570 ns for the wt, and 555.2 ns, 513.2 ns, and 561.5 ns for M654V.

### 2.2 Trajectory Analysis

The consistency of the trajectories was assessed by running Principal Component Analysis on the Cα of the transmembrane helices, representing the largest collective motion of the pores. The projections of the trajectories onto the first two principal components (Supporting Figure S5) were subjected to Linear Discriminant Analysis classifier (Supporting Figure S6), while the overlapping of the conformational space described by the 20 principal components of the individual and concatenated replicas was assessed by calculating Root-Mean-Square Inner Product (RMSIP, Supporting Figure S7) following a previously detailed pipeline.^26^ The analyses of the trajectories were performed using the MDAnalysis Python library^27,28^ and GROMACS^16^ on a >1500 frames trajectory. Each frame corresponded to the structure with the lowest average Cα-RMSD (centroid) of the pore-forming α-helices within the analyzed >1.5 ns trajectory.

#### 2.2.1 Density Maps

Density maps along the *z*-axis (Figure 3a) for POPC molecules were calculated by classifying atoms up to the ester group as the “head” and the remaining aliphatic chain as the “tail”. Similarly, *z*-axis density maps (Figure 3b) were computed based on atomic partial charges (δ) and categorized as follows: acidic (δ < −0.7, excluding basic residues), basic (δ > 0.7, excluding acidic residues), polar (0.3 ≤ |δ| ≤ 0.7), and hydrophobic (|δ| < 0.3). Data presented in Figure 4 were normalized by rescaling the count-based curves so that the sum of their integrals (i.e., the total area under the curves along the *z*-axis) equals 1.

#### 2.2.2 HOLE Analysis

The structure and composition of the TMC1 pore channels were analyzed using HOLE^29^ on the reduced trajectories consisting of 1 frame per ns. The pore channel was identified based on the average coordinates of residues Ser 414 (TM4), Leu 454 (TM5), Thr 528 (TM6), and Asn 583 (TM7), with a maximum radius of 22 Å. Pore profiles were computed by binning radius values along the *z*-axis with a bin size of 0.5 Å.

#### 2.2.3 Dipole Moment Evaluation

The molecular dipole moments were computed for each frame using the formula

1 $$\mathrm{\mu ⃗}\left(D\right)=4.803\sum_{i=1}^{N}q_{i}\vec{r_{i}\;}$$

where *N* is the total number of atoms, *q_i_* is the atomic charge, $\vec{r_{i}\;}$ is the position of the *i*-th atom relative to the center of mass of the system’s partition, and 4.803 is the conversion factor from Å-electron-charge units to Debye. Dipole moments of individual monomers and dimers were calculated relative to their centers of mass. Data are reported as the mean and standard deviation calculated over a 10 ns running average.

## 3 Results

### 3.1 Results: Pore Structure in TMC1 Wild-Type and M654V

Monomer A of TMC1 wt (Figure 2a, top left) exhibited a minimum pore radius (choke point) in an extended region between −5 and 10 Å along the *z*-axis, while monomer B (Figure 2a, top right) displayed the choke point in a narrower region between 0 and −10 Å, with slightly larger radii. Interestingly, the radius profile of monomer B also showed different standard deviation distributions, suggesting a different flexibility of the pore walls, particularly near the choke points. Specifically, monomer A displayed lower fluctuations on the central part of the transmembrane region (−10 Å < *z*-coordinate <10 Å), whereas monomer B showed enhanced flexibility on the cytosolic side (between 0 and −10 Å). Both monomers exhibited increased radii and larger standard deviation at the extramembrane regions (*z*-coordinate < −20 Å and >20 Å), indicative of higher flexibility in these segments.

**Figure 2:**
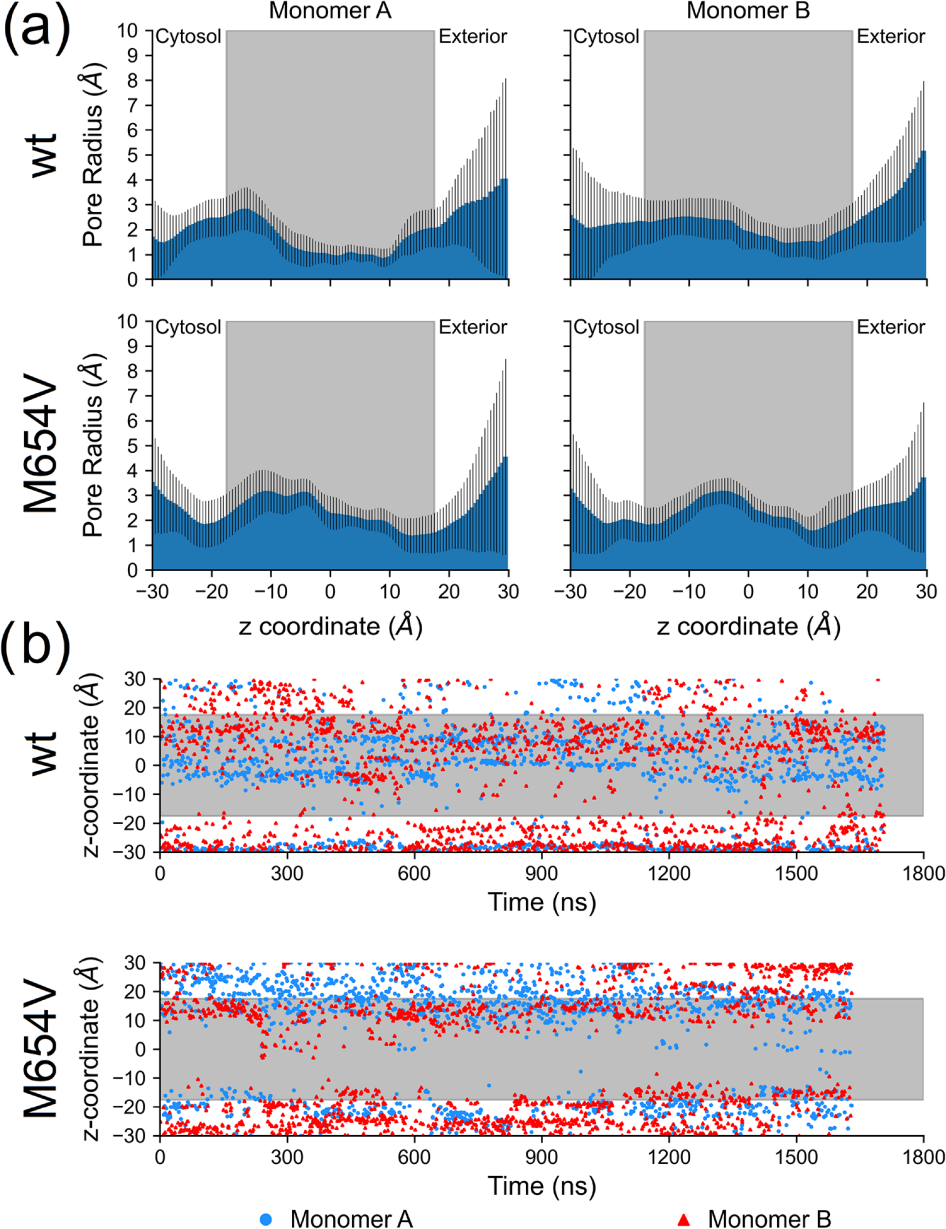
(a) Pore radius for monomers A (left) and B (right) of TMC1 wt (top) and M654V (bottom). Data are calculated on the concatenated trajectory and presented as average ± standard deviation; the gray area indicates the position of the membrane. (b) Time evolution of the *z*-axis position corresponding to the minimum pore radius; the gray area indicates the position of the membrane.

The pore profiles of the two monomers in the TMC1 M654V variant were more similar to each other than in the wt, with a single choke point around 10 Å along the *z*-axis (Figure 2a, bottom panels). Notably, both monomers exhibited a larger pore radius at −10 Å < *z*-coordinate <0 Å compared to their counterparts in the wt, indicating a reshaping of the pore.

Consistent with previous observations, the time evolution of the *z*-coordinate of the choke points (Figure 2b, top) in TMC1 wt indicated that choke points in monomer A were located mainly at *z* ∼ 0 and 10 Å, whereas those in monomer B were found at *z* ∼ −30 and ∼15 Å. Concerning the M654V variant (Figure 2b, bottom), the distribution of choke point positions differed from the wt, but no clear distinction was observed between monomers, as both exhibited choke points at *z* ∼ −25 and 15 Å.

To further assess pore shape and size, we analyzed the distribution of pore center points (Figure 3) in the transmembrane region (−30 Å < *z* < 30 Å) and the corresponding radii across representative frames. Consistent with the higher standard deviations observed in Figure 2a, the center point distribution was broader in TMC1 wt than in M654V, suggesting reduced pore wall flexibility in the mutant. Notably, TMC1 wt showed greater spread in the *xy*-plane, hinting at the existence of multiple extracellular permeation pathways.

**Figure 3:**
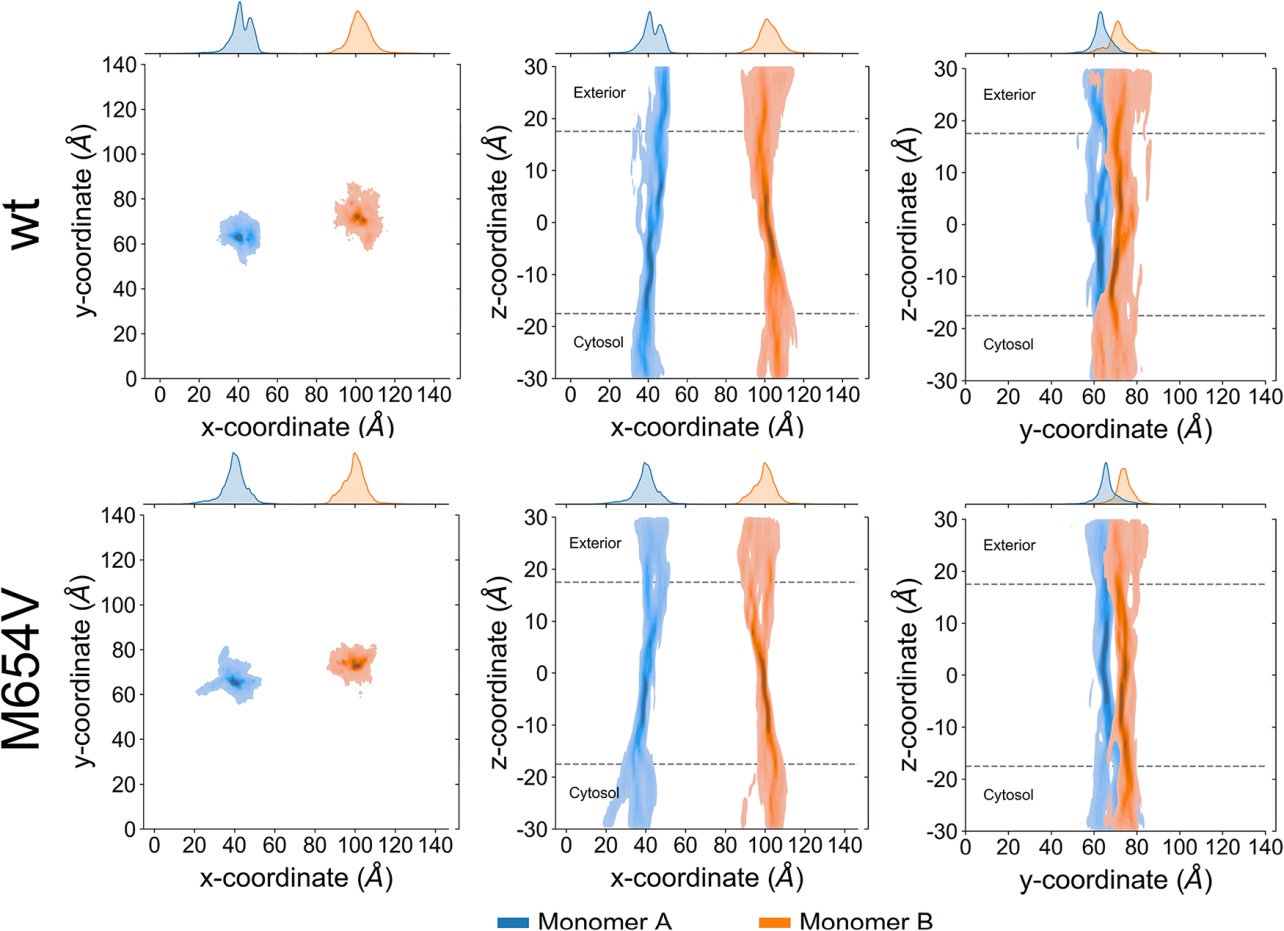
Density maps of the position of the pore centers in monomers A (blue) and B (orange) for TMC1 wt (top panels) and M654V (bottom panels), projected onto the *xy*- (left), *xz*- (center), and *yz*-planes (right). Insets display the marginal distributions along the *x*- or *y*-axis. Data were obtained by fitting the structures to the average channel center points identified by HOLE over the concatenated >1.5 μs trajectories.

Visual inspection of the channel pore structure of TMC1 variants (Figure 3) revealed an extended choke point near the intracellular side in monomer A of the wt, in contrast to monomer B, whose choke point was located in the central part of the membrane (Figure 3, top panels). A similar pattern was observed in the M654V variant, although with a more extended choke point.

### 3.2 Electrochemical Environment in the Pores of TMC1 Wild-Type and M654V

To assess the potential role of lipid molecules as structural components of the pores and to gain further insight into the electrochemical contribution of POPC molecules, phospholipids were subdivided into head groups (comprising choline and glycerol-phosphate moieties) and tails (aliphatic chains). Their occurrence as pore-lining elements was evaluated across the whole trajectories along the *z*-axis (Figure 4a).

**Figure 4:**
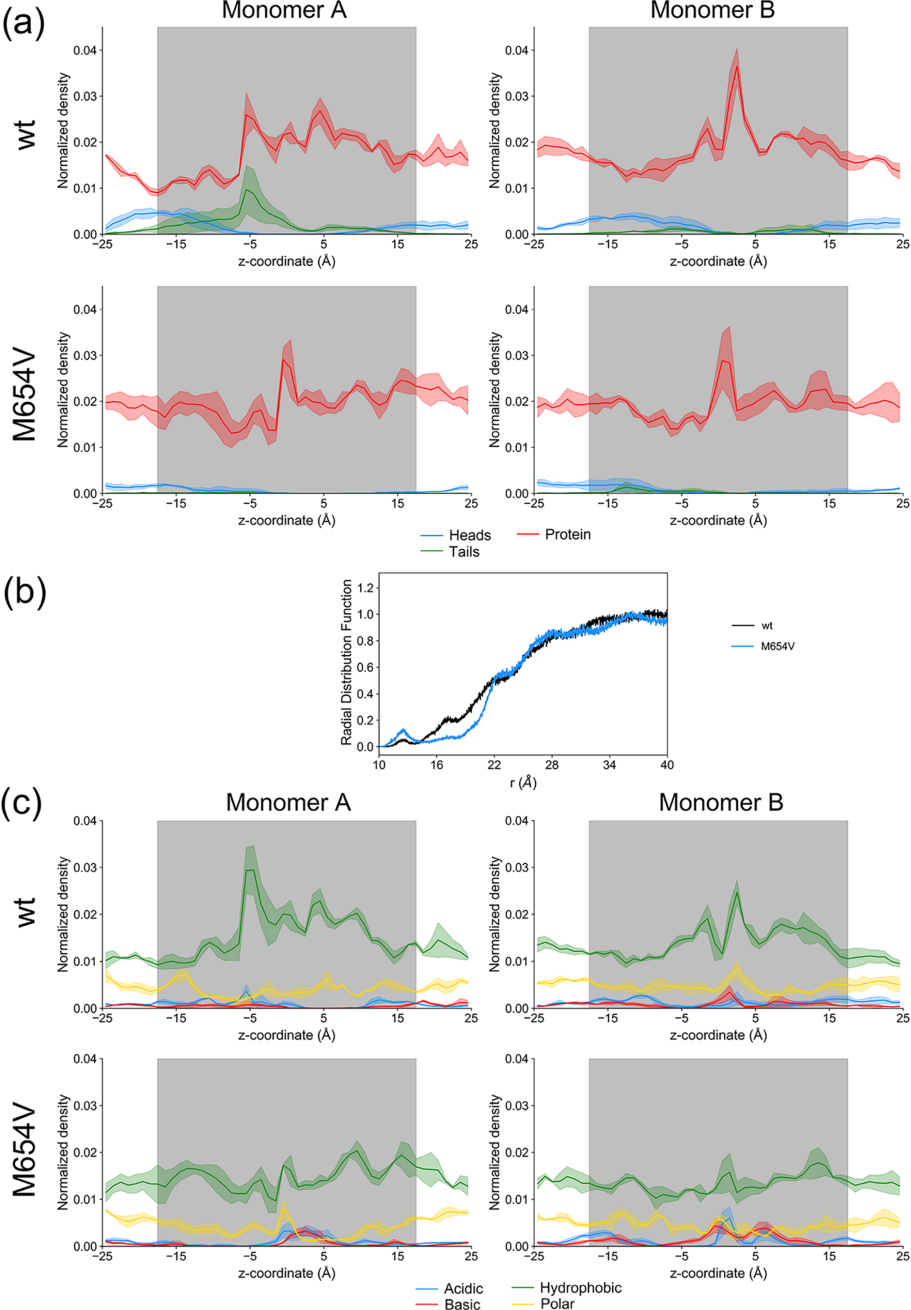
(a) Density along the *z*-axis of the protein (red) and the heads (blue) and tails (green) of POPC molecules as pore-lining residues for monomers A (left panels) and B (right panels) of TMC1 wt (top) and M654V (bottom). The presence of POPC molecules in regions far from the transmembrane domains (*z* < −25 Å and *z* > 25 Å) as pore-lining residues is an artifact due to HOLE calculations and thus not shown. (b) Radial distribution function of POPC molecules along the xy-plane for TMC1 wt (black) and M654V (blue). (c) Fitted density displaying the electrostatic properties of the pore-lining residues for monomers A (left) and B (right) for TMC1 wt (top) and M654V (bottom). Polar atoms are represented in yellow, hydrophobic atoms in green, acidic in blue, and basic in red. The gray areas in (a) and (b) represent the position of the phospholipidic bilayer along the *z*-axis. Data are presented as average ± standard deviation of the three independent replicas for each simulated system and normalized as described in the Methods section.

Interestingly, TMC1 wt (Figure 4a, top panels) featured numerous lipid molecules lining the pore on both the cytosolic and extracellular sides of the transmembrane regions, consistent with previous findings.^12,30^ Specifically, phospholipid head groups contributed to the pore structure across the whole transmembrane span, except for localized regions in the center bilayer core, where tails also played a role. As expected, both monomers showed a bimodal distribution of head groups, with distinct peaks near the membrane periphery, while tails peaked close to the cytosolic side (*z* around −10 Å), with higher occurrence in monomer A.

In contrast, the contribution of membrane lipids to the pore structure in TMC1 M654V was markedly reduced (Figure 4a, bottom panels). Although the head groups distribution qualitatively resembled that of TMC1 wt, their frequency was significantly lower throughout the trajectory. Moreover, POPC tails were nearly absent in the core, hydrophobic region of the membrane, with monomer B presenting only a minor peak at *z* ∼ −10 Å. These findings were corroborated by the radial distribution function (rdf) of POPC molecules around the center of mass of the two proteins projected onto the *xy*-plane (Figure 4b). Interestingly, the peak at ∼13 Å was significantly higher in the variant compared to the wt, while the peak at ∼17 Å exhibited by the wt was almost absent in M654V, indicating distinct lipid packing near the protein surface. Furthermore, the rdf of TMC1 wt exhibited broader fluctuations, possibly reflecting more dynamic lipid–protein interactions, particularly between 16 and 22 Å from the dimer’s center of mass, at odds with M654V, whose rdf rose steeply at ∼22 Å, suggesting a more compact and ordered lipid arrangement at those distances.

To further explore the electrochemical properties of the pores, the identified pore-lining atoms were classified according to chemical type as described in the Methods section, and their distributions along the *z*-axis were analyzed (Figure 4c). All atom classes displayed fluctuating profiles, revealing an alternation of hydrophobic and polar/charged residues along the pore that may form rings acting as selectivity filters, a typical feature of ion channels.

Consistent with previous results, the electrochemical environment of the pores of wt TMC1 monomers (Figures 4c and 5, top panels) was more heterogeneous than that of the M654V variant (Figures 4c and 5, bottom panels), particularly in terms of the distribution of hydrophobic residues within the membrane. Monomer A (Figure 4c, top left panel) of the wt provided a notably more hydrophobic environment in the membrane core, whereas monomer B displayed a larger number of polar residues close to the center of the membrane. In all cases, polar residues were more densely distributed near the membrane edges. Analysis of charged residues revealed a slight predominance of acidic over the basic atoms in both wt monomers (Figures 4c and 5, top panels), with the only exception being the center of the membrane, where a larger number of basic atoms are localized between two clusters of polar and hydrophobic atoms. On the contrary, in M654V (Figures 4c and 5, bottom panels), a more balanced distribution of hydrophobic and polar atoms could be noticed, with peculiar peaks of both acidic and basic residues in proximity to the center of the membrane (0 Å < *z* < 5 Å).

**Figure 5:**
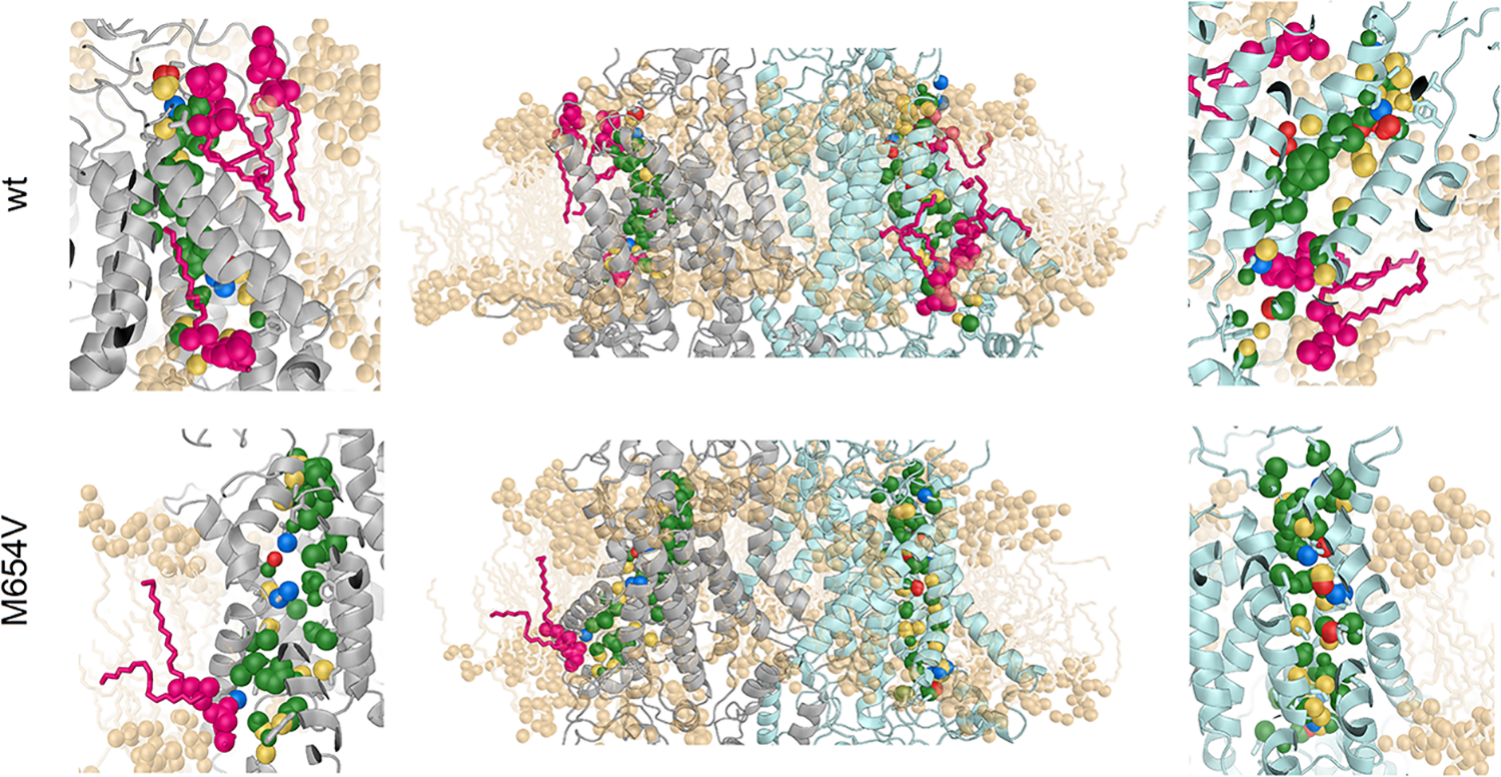
Electrochemical properties of the pore-lining residues in TMC1 wt (top panels) and M654V (bottom panels) variants, shown on the centroid structure of each trajectory. The protein is displayed as cartoons, with monomer A in gray and monomer B in cyan. The side chains of pore-lining residues are depicted as sticks and colored according to their respective monomer, and the tails of lipid molecules are represented by light orange sticks; N, O, and P atoms of the lipid molecule heads are shown as spheres, and pore-forming lipids are colored magenta. Atoms are shown as spheres and colored according to type: polar (yellow), hydrophobic (green), acidic (blue), and basic (red).

Overall, the predominance of acidic and polar residues at the channel entrances, together with the presence of hydrophobic residues interspersed with polar and charged atoms along the pore may influence ion selectivity. Notably, the specific electrostatic patterns differed between monomers, particularly within the transmembrane region, implying that ion permeation could occur independently in each pore of the dimer. In particular, comparison of monomer A between the wt and the M654V variant showed only minor differences, primarily localized on the cytosolic side (Figure 4c, *z* < 5 Å), while monomer B showed more pronounced differences near the membrane center (Figure 4c, −5 Å < *z* < 5 Å), where M654V created a slightly more basic environment around *z* ∼ 0 Å.

### 3.3 Dipole Moments in TMC1 Variants

To investigate differences in the charge distribution between the two TMC1 variants, we computed the dipole moment of the individual monomers (Figure 6a) and the angle formed between the dipole vectors of the two monomers (Figure 6b).

**Figure 6:**
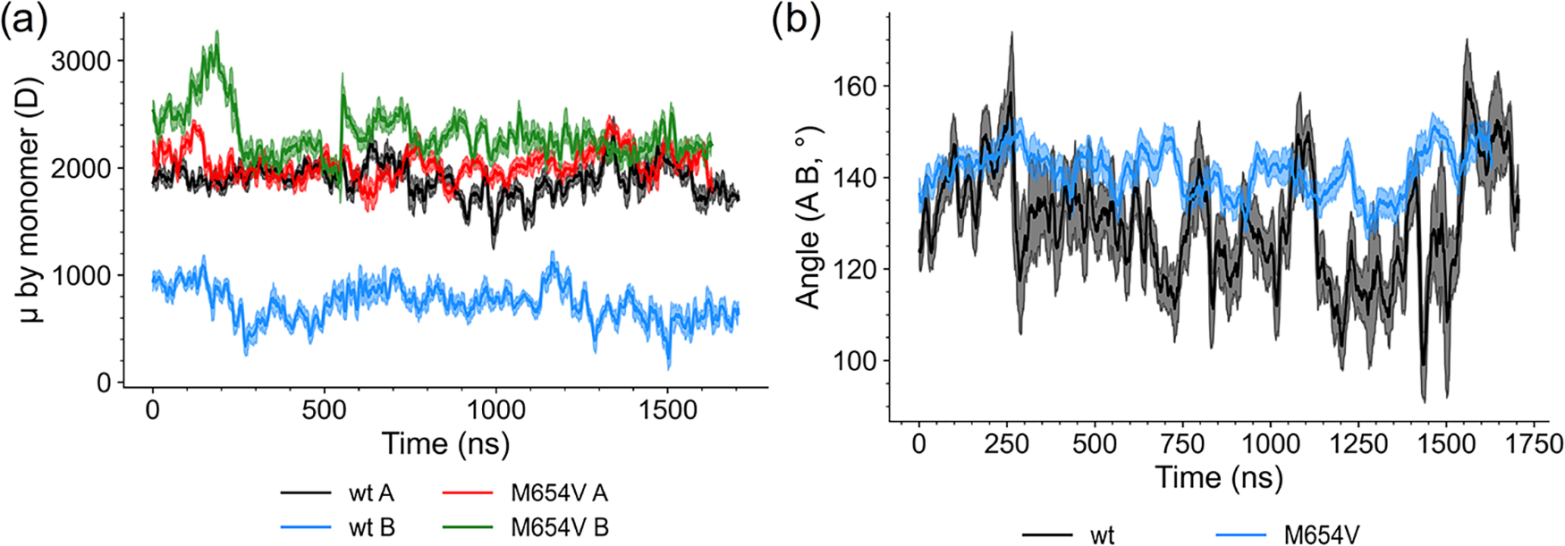
(a) Time evolution of the dipole moment magnitude for monomers A and B of TMC1 wt (black and blue, respectively) and M654V (red and green, respectively). (b) Angle formed between the dipole vectors of the two monomers in TMC1 wt (black) and M654V (blue). Data are presented as the mean (solid lines) and standard deviation (shaded areas) of a running average computed over a 10 ns window.

Interestingly, monomer A exhibited a similar behavior in both variants (Figure 6a), with dipole moments fluctuating around 2000 D throughout the trajectory. In contrast, monomer B showed marked differences: while in the TMC1 wt, the dipole moment oscillated around ∼800 D, in the M654V variant, it oscillated around ∼2200 D. Further differences were found in the angle formed between the dipole vectors of the two monomers (Figure 6b): in the wt, this angle fluctuated between 100° and 150° over time; conversely, the M654V mutant displayed an initial ∼140° angle throughout the trajectory. This, together with the significantly higher fluctuation of the angle exhibited by the wt, suggested an increased structural rigidity in the M654V variant, with a fairly stable relative orientation of the monomers during the simulated time frame.

### 3.4 Effects of the M654V Mutation on Neighboring Residues and Lipids

To gain further insights into the local molecular environment surrounding residue 654, whose mutation impacts both the structure and the electrostatic properties of the channel, as described in previous sections, its contacts with neighboring residues were identified and compared between the two TMC1 variants (Figure 7).

**Figure 7:**
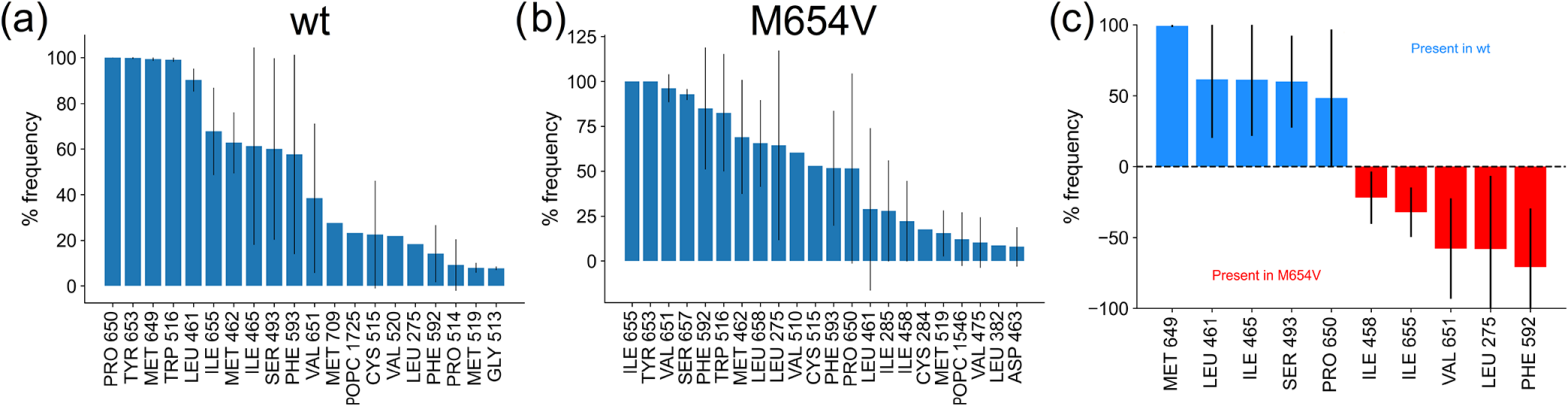
Frequency of residues located within 5 Å from the center of geometry of the side chain of residue 654 in both monomers of TMC1 (a) wt and (b) M654V variants. Frequency is expressed as the percentage of frames in which the distance criterion was satisfied over the total number of frames, and data are presented as average ± standard deviation of the three replicates. For the sake of clarity, only residues with a frequency greater than 5% are shown. (c) Differences in contact frequency between the two variants for residues appearing with >5% frequency. Data for TMC1 wt are shown in blue and for M654V in red. For the sake of clarity, only residues with a frequency variation >10% between the two variants are reported.

Highly persistent interactions (contact frequency >75%) involving residue 654 were fewer in TMC1 wt (4 residues, Figure 7a) than in the M654V variant (6 residues, Figure 7b). This difference reflects the lower average contact frequency of Met 654 in the wild-type protein (23%) compared with Val 654 in the mutant (42%), further supporting the notion of altered local flexibility within the transmembrane region. Notably, a POPC molecule was found close to residue 654 in both cases, although the frequency was almost halved in the M654V variant, suggesting altered interactions with the hydrophobic core of the membrane.

Comparative analysis of contact frequencies (Figure 7c) revealed that in TMC1 wt residue 654 predominantly interacted on the same TM helix with M649 and P650, at odds with the M654V variant, whose primary specific intrahelical contacts were with V651 and I655. Moreover, wt-specific contacts were mainly located between TM5 and TM6 (residues 435–550), while specific contacts in the M654V variant were mainly observed with residues on TM7 (F592), TM2 (L275), and TM5 (I655).

Considering the observed differences in the *z*-coordinate of the choke point between the two variants (Figure 2), with the M654V choke points located closer to residue 654 in both monomers (*z*-coordinate = 15.70 ± 0.56 and 13.30 ± 0.65 Å for monomers A and B, respectively, compared to 9.16 ± 0.69 and 14.68 ± 0.61 Å for the wt), we evaluated whether the mutation affected the local geometry of the pore by calculating the percentage of frames in which the interactors of residue 654 were also identified as pore-lining residues. The results showed that in the M654V variant, this percentage was higher compared to the wt in both monomer A (7.98 vs 7.15%, Table 1) and monomer B (2.51 vs 1.4%, Table 1), thus reinforcing the hypothesis that the mutation shifts the preferential location of the choke point.

**Table 1:** Percentage of Frames in Which Residues Interacting with Position 654 Interactors were Simultaneously Identified as Pore-Lining Residues (Positive), Relative to the Total Number of Analyzed Frames (Total)

variant	monomer	positive/total	frequency (%)
wt	A	122/1707	7.15
wt	B	24/1707	1.40
M654V	A	130/1628	7.98
M654V	B	41/1628	2.51

The differences in intramolecular contacts were also reflected in the distances between residue 654 and the closest residues on all other TM helices (Figure 8a). In general, both monomers of TMC1 wt exhibited larger distances between residue 654 and TM1, TM2, TM7, and TM8, compared to the M654V variant, resulting in an overall ∼0.5 Å difference (Supporting Figure S8), thus suggesting a more compact local environment in the mutant. Notably, the only exception was observed in monomer A, where the distance between residue 654 and TM5 was nearly doubled in M654V.

**Figure 8:**
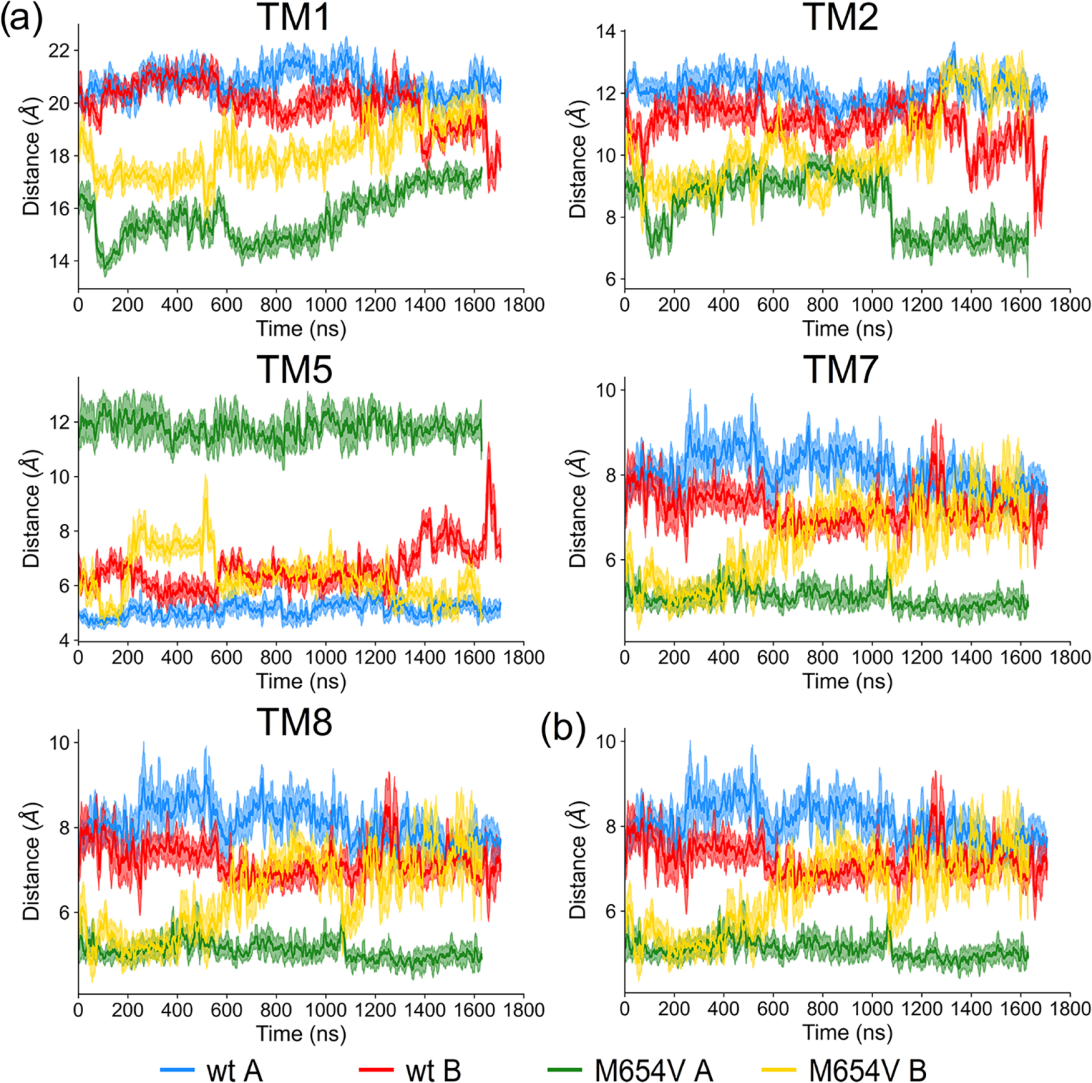
(a) Distance between the center of geometry of the side chain of residue 654 and the closest residue on helices TM1, TM2, TM5, TM7, and TM8. (b) Radius of gyration on the xy-plane of the TM helices in monomers A and B of TMC1 wt (blue and red, respectively) and M654V (green and yellow, respectively), evaluated over the course of the simulation. Data are shown as averages (solid lines) with standard deviations (shaded areas) based on a running average calculated over a 10 ns window.

To assess the overall pore compactness, we monitored the radius of gyration of the TM helices on the *xy*-plane (Figure 8b). Consistent with the interhelical distance data, the average radius of gyration in both monomers of TMC1 wt was ∼0.5 Å larger than in M654V throughout the trajectory, supporting the hypothesis of a more compact transmembrane domain in the mutant.

## 4 Discussion

The structural analysis of the TMC1 wt dimer revealed several notable differences between its monomers. Specifically, the pore profile (Figure 2a) of monomer A exhibited a more elongated and narrower permeation pathway compared to monomer B. Choke points in monomer A were predominantly localized within the cytosolic portion of the membrane and, to a lesser extent, on the extracellular side of the transmembrane region (Figure 2b), while monomer B showed choke points primarily in the central part of the membrane. Additionally, a larger number of POPC molecules were identified as pore-lining residues in monomer A than in monomer B (Figure 4a), suggesting monomer-specific coupling to the membrane. These findings raise the possibility of distinct ion permeation pathways between monomers (Figure 3), particularly near the extracellular-membrane interface. Although these monomer-specific differences, also noted in previous work,^32^ suggest functional independence or distinct permeation states, it should be noticed that the inclusion of the full components of the MET channel may modulate these features under physiological conditions. Moreover, the substantial presence of lipid molecules as pore-lining residues strongly supports a gating mechanism based on the lateral-tension model in which membrane tension regulates channel opening. This interpretation is further reinforced by the radial distribution function of POPC, which indicates a tight lipid–protein coupling likely driven by the influence of the protein surface on local lipid packing. Nevertheless, the prominence of lipids as active contributors to channel function may be highly sensitive to the membrane composition and the presence of TMC1 binding partners, aspects that have not been investigated in this study.

Comparison between TMC1 wt and M654V variant revealed differences in pore architecture, lipid interactions, and electrostatic properties that may collectively contribute to the pathogenic effects associated with hearing loss. The TM helices of TMC1 M654V exhibited a lower average radius of gyration on the *xy*-plane relative to the wt (Figure 8b), accompanied by reduced pore size variability (Figure 2a) and a narrower spatial distribution of pore center points in the TM region (Figure 3). These observations suggest a decreased conformational flexibility of the pore walls for the deafness-associated variant.

Analysis of residue 654 interactors further supports this conclusion. In TMC1 wt, residue M654 is involved in a greater number of contacts with neighboring residues compared to its mutant counterpart. In contrast, the V654 residue was found in closer proximity to TM7 and TM8 helices and displayed a higher frequency of interaction with choke point-associated residues (Table 1), indicating a stronger local coupling that may contribute to pore stabilization while reducing its dynamic range.

The M654V substitution not only altered local residue interactions but also decreased the presence of POPC molecules contributing to pore lining, especially in the bilayer center (Figure 4a). Along with changes in radial distribution functions (Figure 4b), this suggests a weaker lipid–protein coupling in the mutant, particularly at the pore-membrane interface, compared to the more dynamic interplay observed in TMC1 wt, which is likely essential for proper gating behavior. Moreover, a POPC molecule was observed in close contact with residue 654 (Figure 7) in both cases, although such interaction was less persistent in the variant. Nevertheless, the phospholipid appeared closer to TM1, TM2, TM5, TM7, and TM8 compared to native Met 654 in the wt (Figures 8a and S8). This was associated with a lower radius of gyration (Figure 8b), suggesting a more compact structure that may facilitate tighter lipid packing around the transmembrane region.

Electrostatic analysis revealed a more homogeneous charge distribution in the mutant with a relative increase in basic residues, especially in monomer B (Figure 4c), which could influence ion selectivity and permeation. Additionally, the M654V dimer was markedly less flexible than the wt dimer, as shown by the reduced variation in the angle between monomer dipoles (140° in M654V vs 100–150° in wt; Figure 6). While the overall differences in electrostatics and local pore environment between the two variants were modest, even small changes in ion permeation kinetics may severely impact the ultrafast gating behavior required for TMC1’s physiological role in mechanotransduction.^36,37^

## 5 Conclusions

This study provides new molecular insights into the architecture and dynamics of the TMC1 channel and their alterations associated with the pathogenic M654V mutation. All-atom MD simulations of the TMC1 wt dimer supported previous observations of monomer-specific behavior and highlighted a substantial contribution of lipid molecules to pore architecture, suggesting that the channel’s function is tightly coupled to its lipid environment.

On the other hand, the comparison with the M654V mutant variant revealed a series of subtle but functionally significant alterations. The mutation led to increased compactness of the transmembrane domain, reduced conformational flexibility of the pore walls, and a consistent shift in the localization of the channel’s choke point. These effects were accompanied by a lower incidence of lipid molecules lining the pore and a more homogeneous and less dynamic electrostatic profile, factors that could collectively impair the finely tuned gating kinetics required for mechanotransduction.

Altogether, our findings suggest that the molecular basis of hearing loss associated with the M654V mutation may lie in a disruption of the delicate structural and electrostatic balance at the pore–membrane interface. Upon stabilization of local helical packing and decoupling the channel from its lipid environment, the mutation likely interferes with the gating plasticity of TMC1, ultimately compromising its physiological function. These insights underscore the importance of lipid–protein and electrostatic interactions in the function of TMC1 and pave the way for future investigations into therapeutic strategies targeting channel–membrane coupling.

## Supplementary Material



## Notes

Position and dihedral restraints (in kcal/mol Å^2^) employed during NPT equilibration steps and production runs (Table S1); time evolution of the temperature of TMC1 wt (black) and M654V (red) during the NVT equilibration steps (Figure S1); time evolution of the average solvent-accessible surface (SAS) area of POPC molecules of TMC1 wt (black) and M654V (red) during the NVT equilibration steps (Figure S2); time evolution of the density of TMC1 wt (black) and M654V (red) during the NPT equilibration steps (Figure S3); time evolution of the average solvent-accessible surface (SAS) area of POPC molecules of TMC1 wt (black) and M654V (red) during the NPT equilibration steps (Figure S4); projection of the frames of the three >500 ns replicas (R1: blue, R2: orange, R3: green) onto the first two principal components (PC1 and PC2) derived from the covariance matrix calculated on the Cα of the transmembrane helices (Figure S5); linear discriminant analysis of the projection of the frames of the three >500 ns replicas (R1: blue, R2: orange, R3: green) onto the first two principal components calculated on the concatenated >1.5 μs trajectories of TMC1 wt (left) and M654V (right) (Figure S6); Root-Mean-Square Inner Product of the first 20 PC calculated on the three >500 ns replicas vs one another and vs the concatenated >1.5 μs trajectories of TMC1 wt (left) and M654V (right) (Figure S7); and time evolution of the average distance between residue 654 and pore-forming helices (Figure S8) (PDF)
