# Supplementary material for: Altered Pore Composition and Flexibility in a Deafness-Associated TMC1 Variant: Insights from Molecular Dynamics Simulations
Source: ACS Chem Neurosci. 2025 Nov 25;16(24):4602–12. doi: 10.1021/acschemneuro.5c00546 (PMC12715761; doi:10.1021/acschemneuro.5c00546)
Supplement: Supplementary file 1 [file cn5c00546_si_001.pdf]

Altered pore composition and flexibility in a deafness-associated TMC1 variant: insights from molecular dynamics simulations

Davide Zamboni,‖ Valerio Marino,‖ Anna Avesani, Giuditta Dal Cortivo, Gianluca Lattanzi,* and Daniele Dell'Orco*

‖ These authors contributed equally to this work

*Email: gianluca.lattanzi@unitn.it

*Email: daniele.dellorco@univr.it

Supporting information

Supporting Table 1: Position and dihedral restraints (in kcal/mol Å²) employed during NPT equilibration steps.

Step	Backbone	Pore-forming	Sidechains	Phosphorous	Dihedral
**1**	2.40	2.40	1.20	0.96	0.48
**2**	1.20	1.20	0.48	0.48	0.48
**3**	0.48	1.00	0.12	0.01	0.24
**4**	0.12	1.00	0.00	0.00	0.00

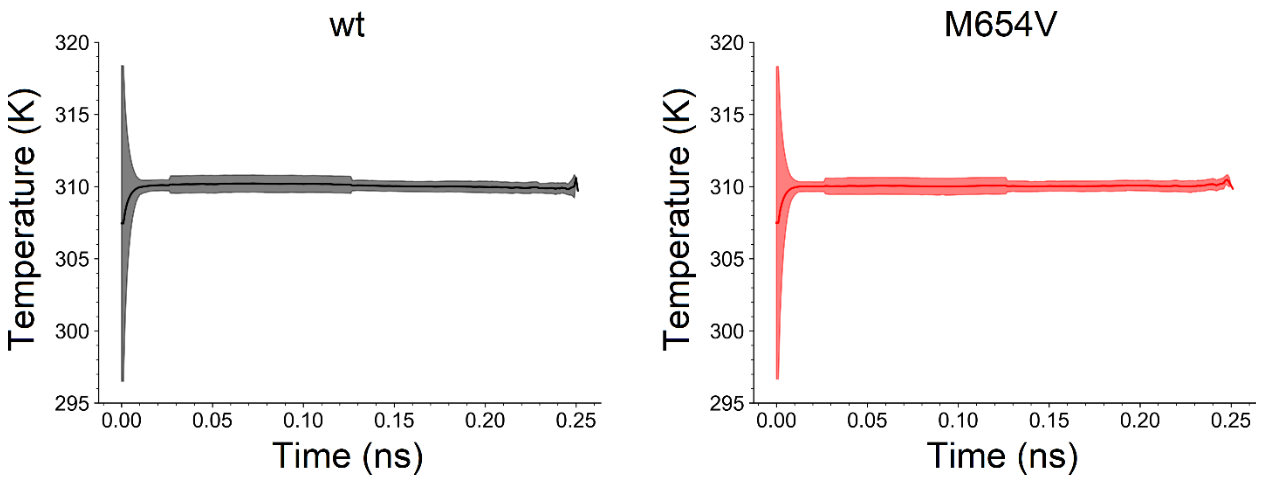

Supporting Figure S1. Time evolution of the temperature of TMC1 wt (left, black) and M654V (right, red) during the NVT equilibration steps; data are presented as average ± standard deviation of the running average over a 10 ns window.

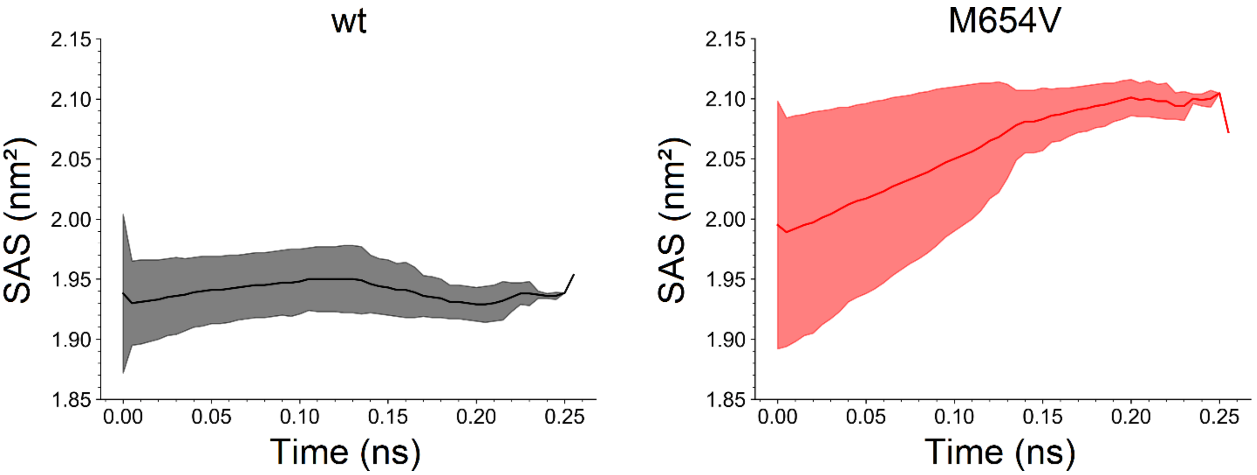

Supporting Figure S2. Time evolution of the average solvent-accessible surface (SAS) area of POPC molecules of TMC1 wt (left, black) and M654V (right, red) during the NVT equilibration steps; data are presented as average ± standard deviation of the running average over a 10 ns window.

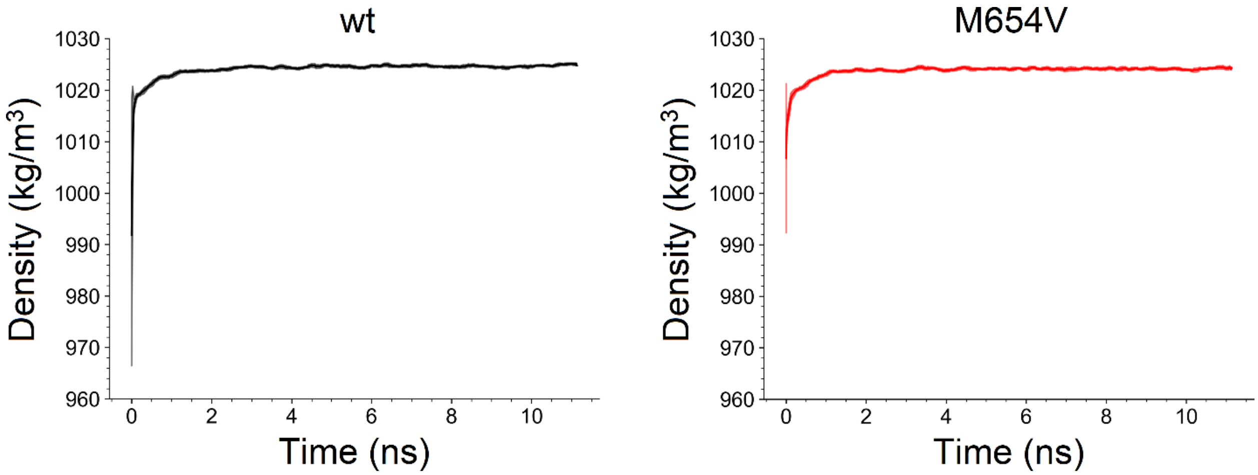

Supporting Figure S3. Time evolution of the density of TMC1 wt (left, black) and M654V (right, red) during the NPT equilibration steps; data are presented as average ± standard deviation of the running average over a 10 ns window.

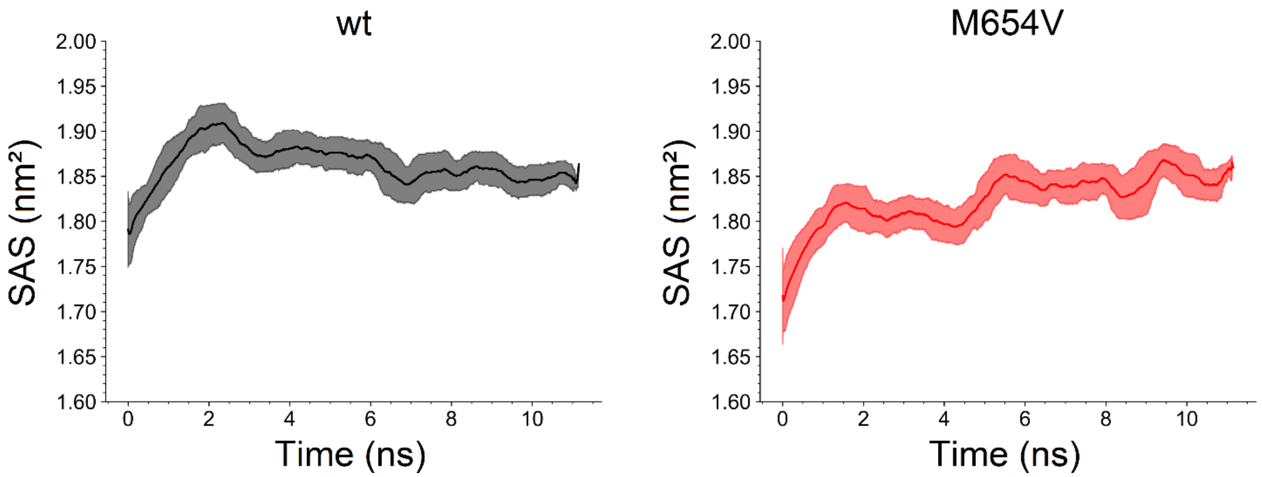

Supporting Figure S4. Time evolution of the average solvent-accessible surface (SAS) area of POPC molecules of TMC1 wt (left, black) and M654V (right, red) during the NPT equilibration steps; data are presented as average ± standard deviation of the running average over a 10 ns window.

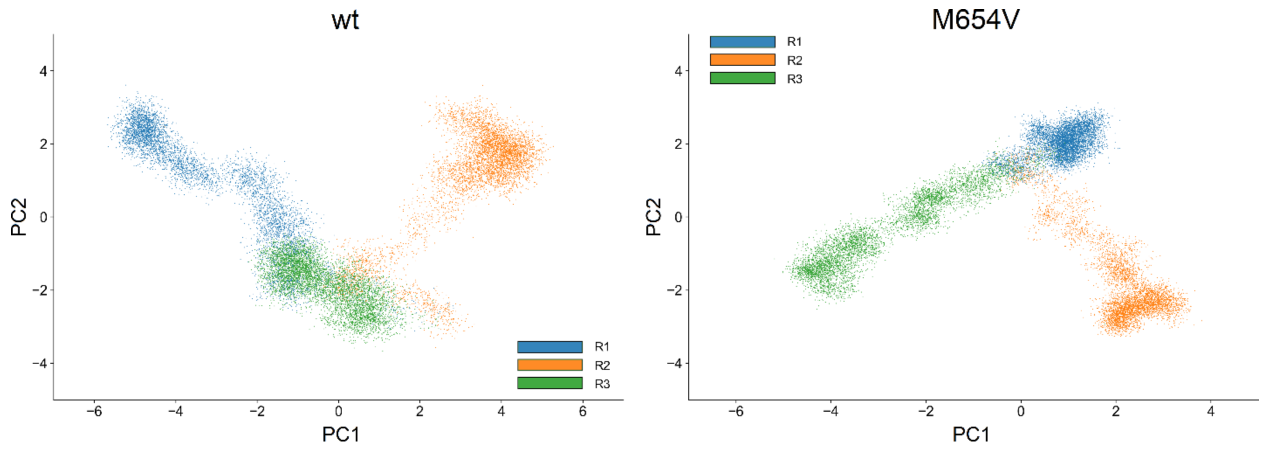

Supporting Figure S5. Projection of the frames of the three >500 ns replicas (R1: blue, R2: orange, R3: green) onto the first two principal components (PC1 and PC2) derived from the covariance matrix calculated on the Cα of the transmembrane helices. PCs were calculated on the concatenated >1.5 µs trajectories of TMC1 wt (left) and M654V (right).

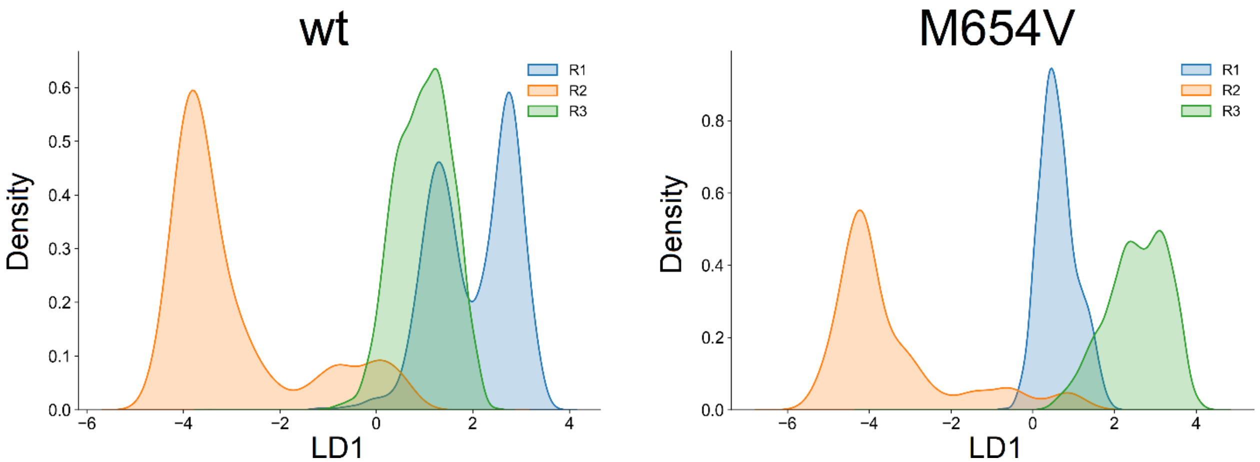

Supporting Figure S6. Linear Discriminant Analysis of the projection of the frames of the three >500 ns replicas (R1: blue, R2: orange, R3: green) onto the first two principal components calculated on the concatenated >1.5 µs trajectories of TMC1 wt (left) and M654V (right).

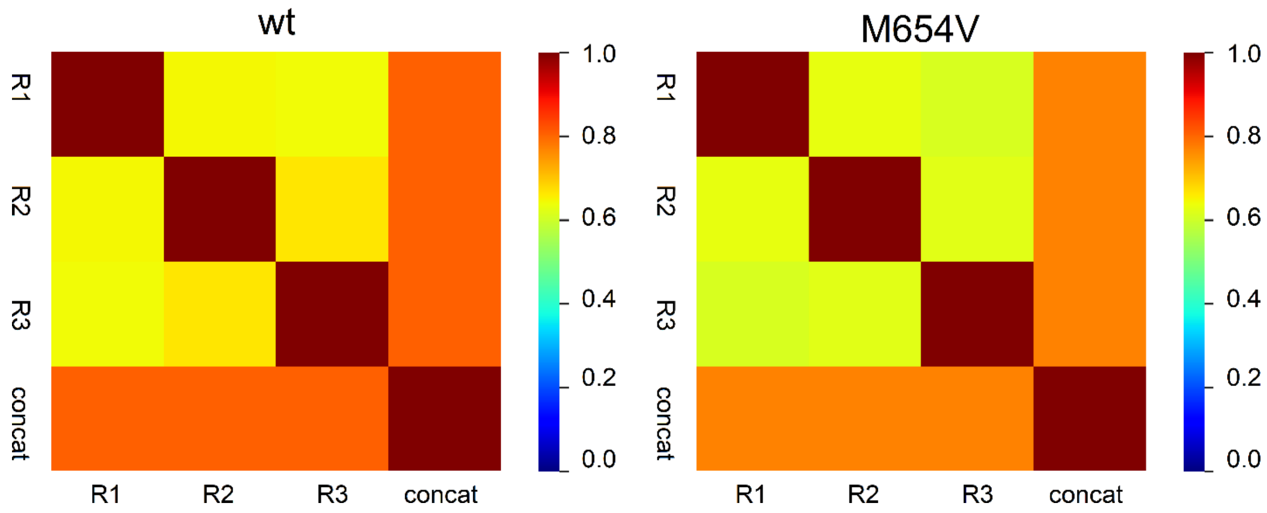

Supporting Figure S7. Root-Mean Square Inner Product of the first 20 PC calculated on the three >500 ns replicas vs one another and vs the concatenated >1.5 μs trajectories of TMC1 wt (left) and M654V (right). RMSIP values, ranging from 0 (orthogonal vectors) to 1 (parallel vectors), are represented in a blue-to-red color scale.

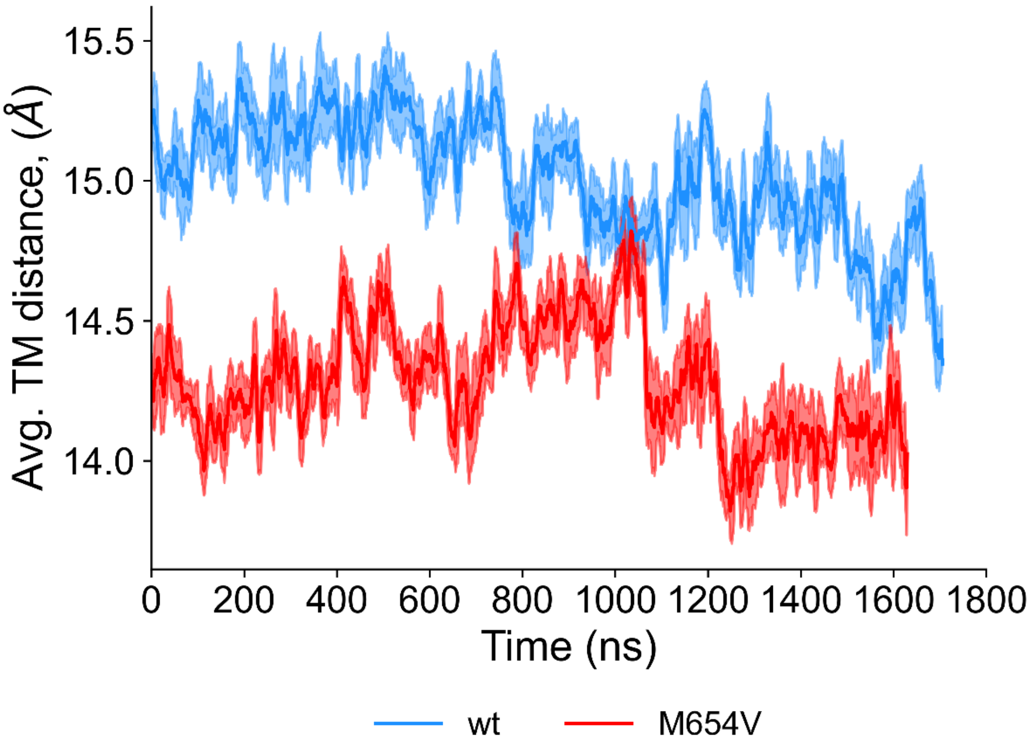

Supporting Figure S8. Time evolution of the average distance between residue 654 and pore-forming helices. Data are presented as average ± standard deviation of the running average over a 10 ns window
